# An Optimized Chickpea Protein Hydrolysate Exerts Long-Term Antihypertensive Effects and Upregulates ACE2 and Mas1 Gene Expression in Spontaneously Hypertensive Rats

**DOI:** 10.3390/foods14203537

**Published:** 2025-10-17

**Authors:** Oscar Gerardo Figueroa-Salcido, Jesús Gilberto Arámburo-Gálvez, Lilian Karem Flores-Mendoza, Giovanni I. Ramírez-Torres, Martina Hilda Gracia-Valenzuela, Edith Oliva Cuevas-Rodríguez, Noé Ontiveros

**Affiliations:** 1 Integral Postgraduate Program in Biotechnology, Faculty of Chemical and Biological Sciences, Autonomous University of Sinaloa, Ciudad Universitaria, Culiacán 80010, Sinaloa, Mexico; oscar.figueroa@uas.edu.mx; 2 Faculty of Nutrition and Gastronomy Sciences, Autonomous University of Sinaloa, Ciudad Universitaria, Culiacán 80010, Sinaloa, Mexico; gilberto.aramburo@uas.edu.mx; 3 Clinical and Research Laboratory (LACIUS, C.N., CONAHCYT National Laboratory, LANIBIOC), Department of Chemical, Biological, and Agricultural Sciences (DC-QB), Faculty of Biological and Health Sciences, University of Sonora, Navojoa 85880, Sonora, Mexico; lilian.flores@unison.mx; 4 Faculty of Physical Education and Sports, University of Sinaloa, Culiacan 80013, Sinaloa, Mexico; giovanni.ramirez@uas.edu.mx; 5 Laboratory for the Research and Detection of Biological Agents and Contaminants (CONAHCYT National Laboratory, LANIBIOC), Instituto Tecnológico del Valle del Yaqui, Tecnológico Nacional de México, Av. Tecnológico, Block 611, Bácum 82276, Sonora, Mexico; martina.gv@vyaqui.tecnm.mx

**Keywords:** chickpea, hypertension, bioactive peptides, antihypertensive, ACE1, ACE2, SHR

## Abstract

Chickpea protein hydrolysates have antihypertensive potential. However, neither the effect of their daily consumption on blood pressure (BP) nor their potential antihypertensive mechanisms has been evaluated. Thus, both the antihypertensive effect of an optimized chickpea protein hydrolysate (OCPH) and its potential mechanisms were assessed in spontaneously hypertensive rats. OCPH (50 mg/kg of body weight) was supplemented daily (5 weeks), BP levels were measured, and mRNA relative levels (angiotensin-converting enzyme-I (ACE1), renin, AT_1_R receptor, ACE2 and Mas1) in the kidneys were determined. BP (systolic, diastolic, and mean) levels were lowered after five days of OCPH supplementation (*p* < 0.05 vs. control group) and the hypotensive effect was up to −39.80 mmHg (*p* < 0.05). Furthermore, the supplementation increased ACE2 (67.30%) and Mas1 (61.1%) mRNA levels (*p* < 0.05 vs. control group). ACE1, renin and AT_1_R receptor mRNA levels were similar between groups (*p* > 0.05). A negative correlation of ACE2 mRNA levels with BP was found (*p* < 0.05). The findings support that OCPH activates the ACE2/Ang-(1–7)/Mas1 pathway of the renin–angiotensin–aldosterone system, maintaining a reduction in BP after daily supplementation. Further studies to evaluate the potential of the OCPH for functional food and nutraceutical development are justified.

## 1. Introduction

Hypertension is the primary risk factor for developing cardiovascular diseases. This chronic disease affects approximately 31.1% of the adult population worldwide [1], leading to strokes and even premature death if not treated properly [2]. The cornerstone treatment involves lifestyle changes, such as increasing physical activity and fruit and vegetable consumption, in combination with pharmacological treatments [3]. In this regard, angiotensin-I-converting enzyme (ACE1) inhibitors are first-line pharmacological agents for hypertension [4]. Other drugs include angiotensin II receptor blockers, calcium channel blockers, and thiazide-like or thiazide diuretics. ACE1 inhibitors and angiotensin II receptor blockers are the most recommended antihypertensive drugs for therapy initiation and can be used alone or in combination with other first-line agents [2]. However, they can trigger side effects such as chronic or dry cough, taste disorders, skin rash, dizziness, and other symptoms [5,6]. Therefore, there is interest in discovering side-effect-free first-line antihypertensive agents. Notably, protein hydrolysates and bioactive peptides can inhibit ACE1 in vitro and reduce blood pressure (BP) in animal models of hypertension [7,8].

Legumes have a high protein content (20–35%) and are rich sources of bioactive peptides [9,10]. Particularly, chickpeas are significant sources of antihypertensive and antidiabetic peptides [11,12,13,14] and the hydrolysis conditions to produce an optimized chickpea protein hydrolysate (OCPH) with high ACE1 inhibitory activity in vitro were recently reported [13]. This OCPH lowered BP in spontaneously hypertensive rats (SHRs), lasting the hypotensive effect for at least seven hours [13]. However, it remains unknown whether a single daily dose of OCPH can trigger a clinically relevant twenty-four-hour antihypertensive effect, as well as the mechanisms underlying this bioactivity beyond ACE1 inhibition.

Renin–angiotensin–aldosterone system (RAAS) is recognized as a pivotal endocrine system that regulates sodium balance, body fluid volumes and BP. RAAS is mainly composed of two opposing axes: the vasoconstrictor (ACE1/AngII/AT_1_R pathway) and vasodilator (ACE2/Ang-(1–7)/Mas1 pathway) axes [15]. ACE1 is a zinc metallopeptidase that catalyzes the conversion of angiotensin I to angiotensin II (AngII), a potent vasoconstrictor that exerts its effect by binding angiotensin type 1 receptor (AT_1_R). This binding induces water and sodium retention, as well as oxidative stress and inflammation. Additionally, ACE1 can hydrolyze the vasodilator bradykinin, contributing to increased levels of BP [16]. The ACE2/Ang-(1–7)/Mas1 axis is known as the counter-regulatory pathway of the ACE1/AngII/AT1 axis, as it induces vasodilation. In this pathway, ACE2 (an ACE1 homolog) catalyzes the conversion of AngII to Ang-(1-7), which binds to the G protein-coupled Mas receptor (Mas1), inducing vasodilatory, anti-inflammatory, and antifibrogenic effects [17]. In hypertension, the ACE1/AngII/AT_1_R pathway is overactivated, contributing to persistently elevated levels of BP [18]. In this sense, evidence suggest that antihypertensive peptides can modulate the expression of key genes of the ACE1/AngII/AT_1_R and/or ACE2/Ang-(1–7)/Mas1 pathways, thereby contributing to their antihypertensive effects. However, there is no evidence about the molecular mechanisms underlying the antihypertensive effects of chickpea hydrolysates. Therefore, the present study aimed to assess the effect of a single daily dose of an OCPH on BP levels and the relative gene expression of ACE1, renin, AT_1_R, ACE2 and Mas1 in the kidneys from SHRs.

## 2. Materials and Methods

### 2.1. Extraction and Protein Concentration

Chickpea seeds (Blanco Sinaloa 92, Granos La Macarena^TM^, Navojoa, México) were milled using a Model 4 Wiley^®^ Laboratory Mill (Thomas Scientific, Swedesboro, NJ, USA), and the protein was obtained [12]. Briefly, chickpea flour was suspended in acetone (1:4 *w*/*v*) and stirred at 500 rpm for 4 h. Precipitates were dried overnight at room temperature and resuspended in distilled water (1:10 *w*/*v*). The pH of the suspension was adjusted to 8.5 (NaOH, 1M), stirred for 2 h (500 rpm), and centrifuged for 10 min (10,000× *g*). The supernatants were collected, and the pellets were washed again. Afterward, supernatants were combined, the pH was adjusted to 4.5 (HCI, 1M), and the mixture was stirred for 2 h (400 rpm). Finally, the solution was centrifuged for 10 min (10,000× *g*) and the pellets were lyophilized and stored at −20 °C.

### 2.2. Chickpea Protein Hydrolysis with Alcalase

OCPH was obtained using optimized hydrolysis conditions [13]. Briefly, protein isolate (15 mg) was suspended in 1 mL of BIS-TRIS propane (20 mM; pH 11). Samples were heated for 30 min (40 °C) and after this time, alcalase (≥2.4 U/g activity, P4860, Sigma-Aldrich, St. Louis, MO, USA) was added (enzyme/substrate ratio = 0.254 U/g) to start the hydrolysis (Thermomixer, Eppendorf™, Hamburg, Germany). The hydrolysis was carried out for 30 min at 40 °C and were stopped by heating (85 °C for 15 min). Afterwards, samples were centrifugated at 10,000× *g* for 10 min and supernatants (hydrolysates) were collected and stored at −20 °C until further use.

### 2.3. Animals

Sample size calculation was determined using the G*Power software (version 3.1.9.7; Heinrich-Heine-Universität Düsseldorf, Düsseldorf, Germany) considering the following parameters: α = 0.05, power = 0.80, effect size d = 1.967 (the effect size was calculated considering the mean and standard deviation differences between the OCPH and control groups from a previous study [13]), expected attrition = 10%. Considering those parameters, a total of fourteen SHRs were required (seven/group). Therefore, fourteen male SHRs (11 weeks old and weighing 220–250 g) were used in the present study (National Autonomous University of Mexico, Cell Physiology Institute). The SHRs were housed in plastic cages with stainless steel lids. Room temperature was controlled (23 ± 2 °C) and 12 h light/dark cycles were established. Water and food (LabDiet^®^ 5001, Richmond, IN, USA) were available ad libitum.

### 2.4. Blood Pressure Assessment

**Figure 1:**
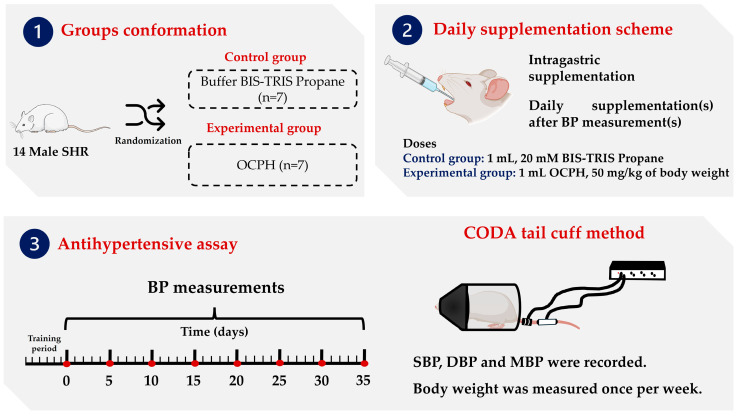
General workflow employed to assess the antihypertensive effect of OCPH. Acronyms: OCPH, optimized chickpea protein hydrolysate; BP, blood pressure; SBP, systolic blood pressure; DBP, diastolic blood pressure; MBP, mean blood pressure.

Figure 1 shows the BP assessment general methodology. SHRs were randomly assigned to two groups (seven rats per group). The treatments (BIS-TRIS propane solution (20 mM) and OCPH (50 mg/kg of body weight)) were administered daily for 5 weeks using sterile feeding tubes (18 GA × 75 mm, Instech Laboratories, Inc., Plymouth Meeting, Montgomery, PA, USA). Before treatment administration, a one-week training period was established to accustom rats to the procedure and guarantee the measurements’ reliability. BP was measured using a tail-cuff method (CODA tail cuff, Kent Scientific, Torrington, CT, USA). Systolic blood pressure (SBP), diastolic blood pressure (DBP), and mean blood pressure (MBP) levels were measured. MBP was calculated using the following Equation (1):

(1) $$\;MBP=DBP+\frac{1}{3}\;(SBP-DBP)$$

BP was recorded before the first treatment administration (time 0) and every 5 days (before vehicle or OCPH administration). The procedure started at the same time (~9 am), keeping the order of the SHRs evaluated. Before BP evaluations, SHRs were kept at 38 °C (5–10 min) to make the pulsations of the tail artery detectable. BP values were reported as the means of at least five reliable BP measurements. The SHRs’ body weight was measured weekly at the same time. The same operator performed all measurements in a quiet environment. At the end of the antihypertensive protocol, rats were anesthetized and euthanized by pentobarbital administration (50 mg/kg). Organs were extracted from the rats, weighed and stored at −80 °C until further use. The organ index was calculated using the following equation:

$$Organ\;index=\;\frac{Organ\;weight}{Rat\;weight}*100$$

### 2.5. RNA Extraction and Quantification

In line with previous studies [19,20,21], five rats per group were randomly selected for gene expression assays. Total RNA was isolated from kidney tissues using TRIzol reagent (Invitrogen, Waltham, MA, USA). Briefly, kidney tissues were homogenized (Polytron^®^ PT 10-35 GT) in 500 μL of TRIzol reagent. Afterwards, 100 μL of chloroform were added to the mixtures and incubated for 3 min at room temperature. Samples were centrifuged at 14,000× *g* (HERMLE Z 216 MK) for 15 min at 4 °C and the upper aqueous phase was collected. The aqueous phase was mixed with 250 μL of isopropanol and the mixtures were incubated for 10 min at room temperature, followed by centrifugation at 14,000× *g* for 10 min at 4 °C. The supernatants were discarded, and the pellets were washed twice with 500 μL of 75% ethanol (vortexed and centrifuged at 5000× *g* for 5 min at 4 °C). Washed RNA pellets were air-dried, and RNA concentration was determined using a NanoDrop 2000 spectrophotometer (ThermoFisher Scientific, Waltham, MA, USA). RNA purity (A_260_/A_280_ and A_260_/_230_ ratios) and integrity (agarose gel electrophoresis) were determined.

### 2.6. Relative Gene Expression Analysis

cDNA was synthesized from 1 μg of RNA using the ABScript II cDNA First-Strand Synthesis kit (ABclonal Technology Co., Ltd., Wuhan, China, RK20400) according to the manufacturer’s instructions. q-PCR was performed to determinate the gene expression levels of ACE1, renin, AGTR1a, ACE2 and Mas1 using a MyGoPro PCR instrument (MyGo PCR, Middlesbrough, UK) and TaqMan™ Fast Advanced Master Mix (Applied Biosystems, Thermo Fisher Scientific, Waltham, MA, USA). β-actin was used as the housekeeping gene. The following TaqMan probes were used: Rn00561094_m1 (ACE), Rn02586313_m1 (ren), Rn02758772_s1 (Agtr1a), Rn01416293_m1 (ACE2), Rn00562673_s1 (Mas1), and Rn00667869_m1 (Actb). All reactions were carried out in duplicate. q-PCR amplification conditions were performed as follows: 50 °C for 2 min, 95 °C for 2 min and 40 cycles at 95 °C for 3 s and 60 °C for 30 s. The relative gene expression was assessed using the 2^−∆∆Ct^ method [22].

### 2.7. Statistical Analysis and Ethical Aspects

Statistical analyses were performed using GraphPad Prism 8.0. (GraphPad Software, San Diego, CA, USA). Data distribution (Shapiro–Wilk test) and homoscedasticity (Barlett’s test) were assessed. The data were expressed as the mean and standard deviation. Differences in BP, body weight, organ index and relative gene expression between groups were determined using an unpaired *t*-test. A repeated-measures ANOVA followed by a Dunnett test was utilized to compare BP values at each time point to basal BP (time 0). Pearson’s correlation coefficient was utilized to determine the correlation of relative gene expression with BP levels. A *p*-value < 0.05 was considered statistically significant. The study protocol was approved by the Ethics Committee of the Autonomous University of Sinaloa (CE-UACNYG-2015-SEP-001).

## 3. Results and Discussion

### 3.1. Optimized Chickpea Protein Hydrolysate Chronic Supplementation Has No Impact on Body Weight but Reduces Kidney Weight

**Figure 2:**
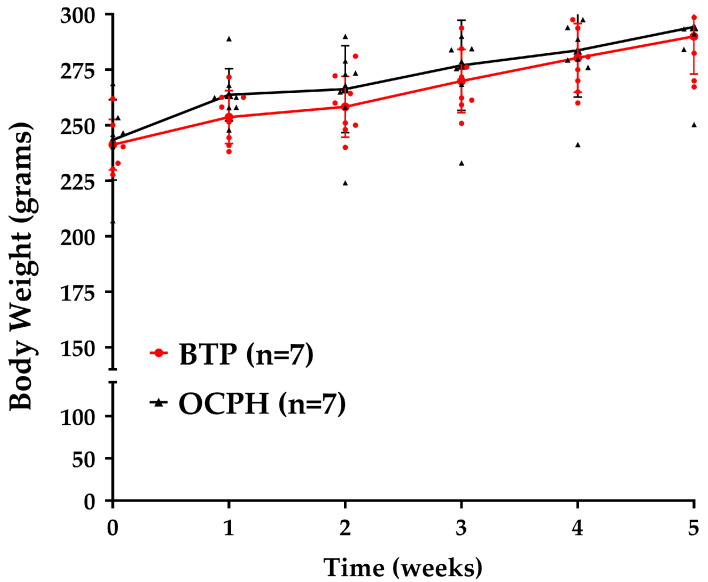
Effect of OCPH supplementation (n = 7) and BTP (buffer BIS-TRIS Propane) (n = 7) on body weight. Data are presented as the mean and standard deviation. Red dots (BIS-TRIS Propane buffer group) and black triangles (OCPH group) represent individual weight values for each SHR evaluated. Raw data are shown in Supplementary Material S1.

Recent studies have shown that CPHs can inhibit ACE1 in vitro and that their supplementation can reduce BP in SHRs for at least seven hours [12,13]. However, there is limited data regarding the dose and time interval between successive administrations of CPHs to help control hypertension. Our results indicate that daily OCPH supplementation for 5 weeks neither changed the feces’ physical characteristics, such as consistency, shape, and color, nor the perceived patterns of supplying water and food. Furthermore, the supplementation did not change body weight between the OCPH and control groups at any time evaluated (*p* < 0.05) (Figure 2). These findings suggest that OCPH supplementation has no weight-loss potential; in line with others that have reported similar findings even after supplementing protein hydrolysates for more than 5 weeks [23,24].

**Figure 3:**
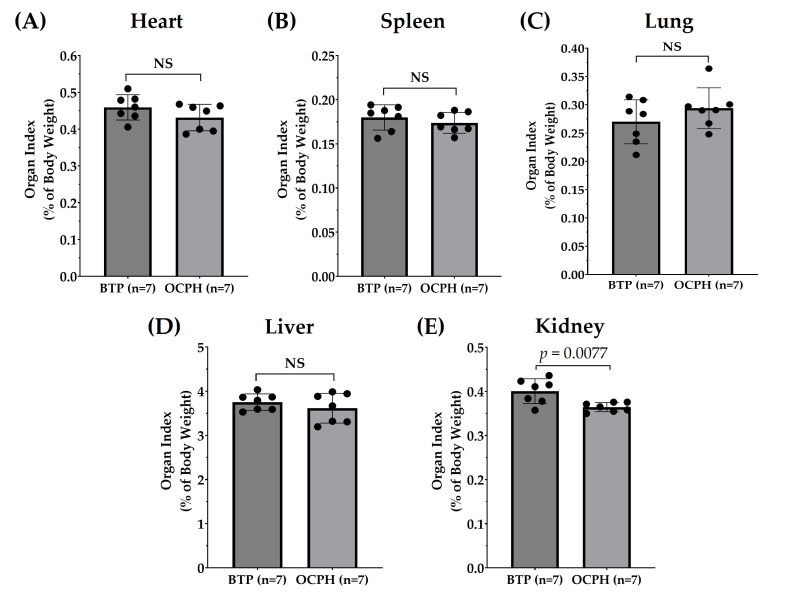
Effect of OCPH (n = 7) and vehicle (buffer BIS-TRIS Propane) (n = 7) supplementation on organ weight. (**A**) heart; (**B**) spleen; (**C**) lung; (**D**) liver; and (**E**) kidney organ weight. Data are presented as the mean and standard deviation. Acronyms. BTP, buffer BIS-TRIS Propane; OCPH, optimized chickpea protein hydrolysate; NS, not significant. Black dots represent individual organ index values for each rat evaluated. Raw data are shown in Supplementary Material S1.

Organ weight changes are frequently used as indicators of hypertension-induced organ damage [25,26]. In our study, chronic supplementation with OCPH did not change the weights of the heart, spleen, lung, and liver (*p* > 0.05) (Figure 3A–D), which is in line with the reported by others, who have shown that hydrolysate supplementation does not affect organ weight despite antihypertensive effects [26,27]. Notably, our data show that kidney weight was reduced in the OCPH group compared with the control group (−9.02%, *p* < 0.05) (Figure 3E). It has been reported that antihypertensive peptide supplementation reduces kidney weight in SHRs, potentially by attenuating hypertension-induced renal injury [18,25]. In fact, hypertension causes inflammation, fibrosis, and hypertrophy, impairing renal function [28]. The central role of the kidneys in regulating BP was demonstrated when kidneys from SHRs were transplanted to normotensive ones, resulting in the latter developing hypertension [29]. Overall, our findings suggest that the reduction in kidney weight in the OCPH group may be attributed to a potential protective effect against hypertension-induced renal injury. However, histological evaluations are necessary to assess typical morphological alterations in the kidneys related to hypertension, such as arteriolar wall thickening and glomerulosclerosis, to support the notion that OCPH could be a potential protector against hypertension-induced renal injury.

### 3.2. Optimized Chickpea Protein Hydrolysate Supplementation Triggers a Sustained Antihypertensive Effect

**Figure 4:**
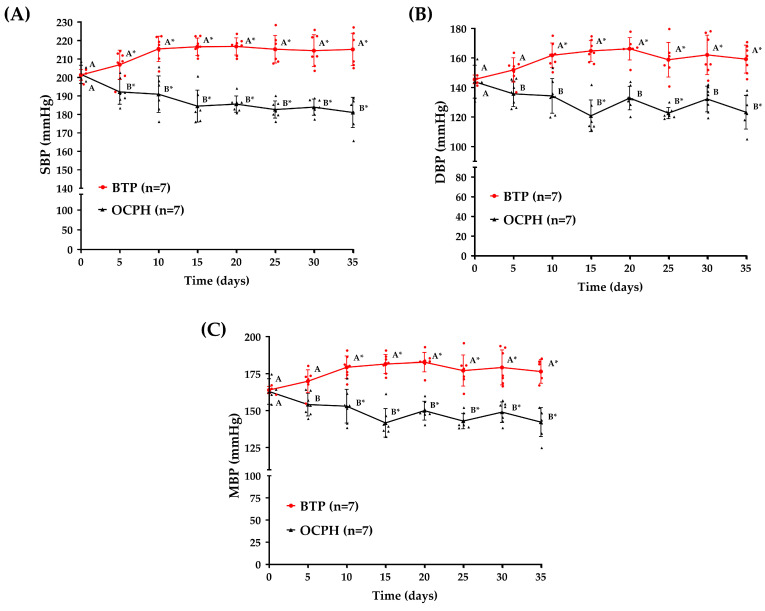
Long-term effect on BP in SHR supplemented with BTP (n = 7) (1 mL, 20 mM) or OCPH (n = 7) (50 mg/kg of body weight). (**A**) SBP, (**B**) DBP and (**C**) MBP. Data are presented as the mean and standard deviation. Vertically, different letters across treatments indicate statistical differences (*p* < 0.05). Horizontally, the asterisk indicates statistical differences in each time point compared to basal values (*p* < 0.05). Red dots (BTP group) and black triangles (OCPH group) represent individual BP values for each rat evaluated (Individual BP data for each rat are shown in Supplementary Figure S1). Raw data, changes in blood pressure (Δ) with 95% confidence intervals and exact *p*-values are shown in Supplementary Material S1, Supplementary Material Figure S2 and Supplementary Material Table S1, respectively.

Daily OCPH supplementation lowered SBP, DBP, and MBP (*p* < 0.05 vs. control group) (Figure 4A–C). Compared to baseline (time 0), OCPH supplementation achieved MBP reductions ranging from −8.81 mmHg (5.41%) to −21.24 mmHg (13.03%) (Days 5 and 15, respectively) (Figure 4C). Similar effects were observed for SBP (from −9.50 mmHg (4.48%) to −20.16 mmHg (10.01%)) (Figure 4A) and DBP (from −8.21 mmHg (5.70%) to −23.12 mmHg (16.06%)) (Figure 4B). This decrease in BP is clinically relevant since lowering SBP by at least 5 mmHg reduces the risk of adverse cardiovascular events by 10% [30]. Notably, BP levels were similar between the control and OCPH groups at time 0 (*p* > 0.05) (Figure 4A–C) and significantly increased compared to time 0 in the control group (*p* < 0.05) (Figure 4A–C). SHR is a model characterized by an increase in BP, which starts around the 6th week of life and usually becomes stabilized around the 12th week [31]. In the present study, we used 11-week-old male SHRs, which explains the increase in BP compared to time 0 in the control group. The data also show that OCPH can prevent this BP increase, triggering a sustained hypotensive effect throughout the experiment (Figure 4A–C). For instance, the control group showed a 13.44 mmHg increase in MBP from day 0 (164.77 ± 3.13 mmHg) to day 25 (178.21 ± 10.14 mmHg) (*p* < 0.05) (Figure 4C), but the OCPH group showed a decrease in MBP of −19.98 mmHg in the same period of evaluation (from day 0 = 163.02 ± 8.66 mmHg to day 25 = 143.04 ± 5.25 mmHg) (*p* < 0.05) (Figure 4C). This means a difference of −35.17 mmHg in MBP between the OCPH and control group at day 25 (Figure 4C). Certainly, ACE1 inhibitory peptides can reach the bloodstream, but they become hydrolyzed by plasma proteases, losing their inhibitory effect around 1-h post-supplementation. Despite this notion, some studies suggest that the hypotensive effect of a single administration of protein hydrolysates is sustained for more than 8 h, but it is lost after 24 h [26,32,33,34]. This suggests that OCPH peptides could not only reach the bloodstream and inhibit ACE1, resulting in an acute reduction in BP, but could also reach tissues other than blood, triggering a clinically relevant twenty-four-hour hypotensive effect in SHRs, in line with our findings (Figure 4A–C). Indeed, antihypertensive peptides can promote biological effects at the local level, reducing ACE1 activity in tissues such as the kidneys, lungs, and heart [26]. Additionally, they could enhance endothelium-dependent vasorelaxation in aortic rings, papillary muscle, and mesenteric arteries, and could reduce vascular inflammation [35,36]. Overall, our data support that daily supplementation with OCPH triggers a sustained and clinically relevant hypotensive effect in SHRs.

Interestingly, previous studies reported a larger CPH-induced antihypertensive effect than the one informed in the present research (−20.16 mmHg vs. −61.41 mmHg [12] and −20.16 mmHg vs. −47.35 mmHg [13]), but those studies were carried out under an acute supplementation scheme [12,13]. Others evaluated the daily administration of antihypertensive synthetic peptides (SBP reduction from −20 to −25 mmHg) [37,38] or hydrolysates from food sources other than chickpeas (SBP reduction from −9 to −35 mmHg), [12,26,32,33], reporting SBP reductions to a similar extent as those reported in the present study. It should be noted that the OCPH dose (50 mg/kg of body weight) used in the present study is from 4- to 30-fold lower than those used by others who tested protein hydrolysates other than chickpea [26,32,33]. This dose translates to a human dose of 8.11 mg/kg/day (calculated using the human equivalent dose available on DoseCal platform [39]), which accounts for 567.57 mg/day for an individual weighing 70 kg. To the best of our knowledge, this is the first in vivo study to assess the effect of daily OCPH supplementation on BP.

### 3.3. Optimized Chickpea Protein Hydrolysate Supplementation Increases ACE2/Ang-(1–7)/Mas1 Pathway Expression in the Kidneys

**Figure 5:**
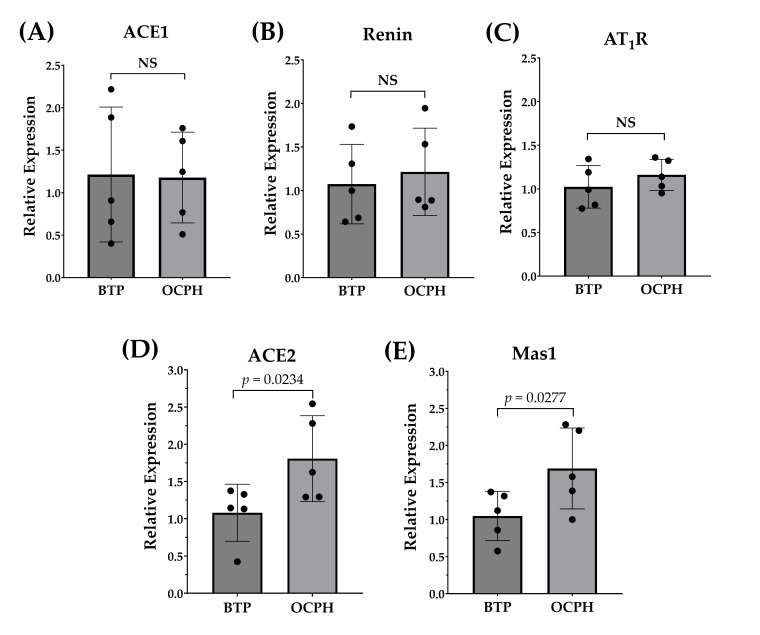
Long-term effect of OCPH supplementation on the gene expression of RAAS components in SHRs (n = 5/group). (**A**) ACE1; (**B**) renin; (**C**) AT_1_R; (**D**) ACE2; and (**E**) Mas1 relative expression. Data are presented as the mean and standard deviation. Black dots represent individual relative expression values for each rat evaluated.Acronyms: BTP, buffer BIS-TRIS Propane at 20 mM; OCPH, optimized chickpea protein hydrolysate (50 mg/kg of body weight). Raw data are shown in Supplementary Material S1.

The relative gene expression of RAAS components is shown in Figure 5. Compared to the control group, OCPH supplementation did not significantly change the expression of ACE1, renin, and AT_1_R (*p* > 0.05) (Figure 5A–C), but it significantly upregulated the gene expression of ACE2 (67.3%) and Mas1 (61.1%) (*p* < 0.05) (Figure 5D,E). These findings suggest that OCPH supplementation upregulates the ACE2/Ang-(1–7)/Mas1 pathway without affecting the expression of major components of the ACE1/AngII/AT_1_R pathway. Similarly, supplementation with a chicken muscle hydrolysate or with the tripeptide IRW, as well as the administration of egg white-derived peptides, can reduce BP and increase ACE2 expression in SHRs [27,36], while not effecting the plasma levels of ACE1 or its expression in the aorta [27]. Others reported that supplementation with peptides derived from *Spirulina platensis* or rapeseed significantly upregulates ACE2 and Mas1 expression in SHRs, but that such supplementation downregulates the expression of ACE1 and AT_1_R [19,20]. Overall, evidence indicates that hydrolysates/peptides can differentially modulate RAAS components, thereby contributing to their antihypertensive effects in vivo.

**Figure 6:**
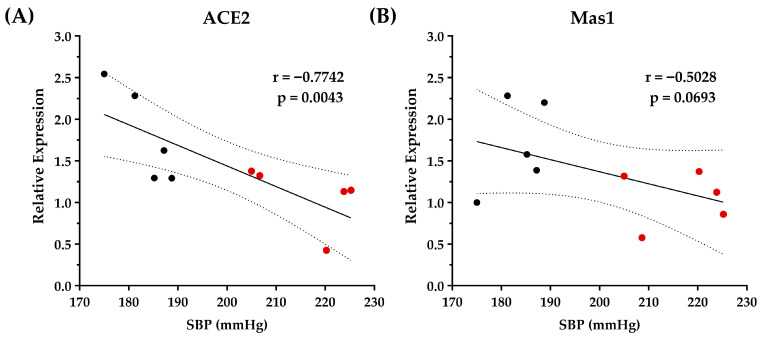
Correlation between relative gene expression levels of ACE2 (**A**) and Mas1 (**B**) with BP in SHRs supplemented with buffer BIS-TRIS Propane (n = 5) (1 mL, 20 mM) or OCPH (n = 5) (50 mg/kg of body weight). Acronyms: SBP, systolic blood pressure. Red dots (BTP group) and black dots (OCPH group) represent individual BP values for each rat evaluated. Correlation between relative gene expression and SBP was determined using Pearson’s correlation coefficient. A *p*-value < 0.05 was considered statistically significant.

ACE2/Ang-(1–7)/Mas1 pathway activation has emerged as an attractive strategy for the management of hypertension [40]. In SHRs, ACE2 overexpression is reported to reduce BP [41]. In line with this, we found a significant negative correlation between upregulation of ACE2 gene expression and SBP (Figure 6A), DBP, and MBP (Figure S3), indicating that OCPH-induced upregulation of the ACE2 gene expression is fundamental for lowering BP levels in SHRs. In the case of Mas1, no statistically significant correlation was detected (*p* > 0.05), but a strong tendency to a negative correlation with BP levels was observed (SBP: r = −0.5028, *p* = 0.0693 (Figure 6B), DBP: r = −0.3615, *p* = 0.1523 and MBP: r = −0.4069, *p* = 0.1216 (Figure S3)). Evidence indicates that OCPH can inhibit ACE1 in vitro [13], but an effect on the ACE1 gene expression was not observed in the present study (*p* > 0.05). Taking this into account, it is hypothesized that OCPH inhibitory peptides may reach the bloodstream, inhibiting ACE1 at the protein level and limiting AngII generation, but without altering ACE1 gene expression in the kidneys. Notably, AngII binding to AT_1_R has been shown to downregulate ACE2 gene expression through the AT_1_R–ERK/p38 MAP kinase pathway [42], whereas AT_1_R antagonists (e.g., losartan) can upregulate ACE2 expression [43]. In this context, OCPH peptides may reduce AngII production due to ACE1 inhibition, thereby alleviating the suppressive effect on the ACE2/Ang-(1–7)/Mas1 axis. This would enable high Ang-(1-7) levels, resulting in vasodilation, anti-inflammatory, and anti-fibrotic effects, which could explain the decrease in kidney weight after OCPH supplementation (*p* < 0.05 vs. control group) (Figure 3E).

We should acknowledge that further studies determining AngII, Ang-(1–7), ACE1, and ACE2 at the protein level are needed to better support the underlying mechanisms of the antihypertensive effect of the OCPH. In this context, the tripeptide IRW is known as an ACE2 activator that ameliorates vascular inflammation and endothelial dysfunction by upregulating the Akt/eNOS signaling and reducing interleukin 6, monocyte chemoattractant protein 1, and cyclooxygenase levels in SHRs, but without affecting the expression, blood concentration, and activity of ACE1 [36]. Therefore, the effects of the OCPH supplementation on vascular inflammation, oxidative stress, and endothelial dysfunction are topics that deserve further research to gain a more comprehensive understanding of the antihypertensive mechanism of the hydrolysate. Finally, evaluating the association between the amino acids generated and hypertension, characterizing the OCPH peptides, and applying bioinformatic approaches, such as molecular docking and molecular dynamics, could provide valuable insights into the mechanisms underlying the antihypertensive effects of OCPH. For instance, peptides derived from food protein hydrolysis have been shown to exhibit strong binding affinities to ACE1, renin, and AT_1_R, which may contribute to their antihypertensive activity [44,45].

## 4. Conclusions

This is the first in vivo study to evaluate the long-term antihypertensive effect of a single daily dose of an OCPH. The data support that the hydrolysate evaluated can trigger a clinically relevant long-term hypotensive effect. This effect is potentially mediated by upregulating the ACE2/Ang-(1–7)/Mas1 pathway, suggesting that the OCPH is a potential activator of the vasodilatory axis of RAAS. Further studies assessing Ang-(1–7), ACE2 and Mas1 at the protein level are needed to establish that OCPH is an activator of the ACE2/Ang-(1–7)/Mas1 pathway. Overall, the findings highlight the potential of the OCPH as a nutraceutical or ingredient for functional food development, bringing it closer to entering clinical trials.

## Data Availability

The original contributions presented in this study are included in the article and Supplementary Material. Further inquiries can be directed to the corresponding authors.

## Supplementary Materials

The following supporting information can be downloaded at: https://www.mdpi.com/article/10.3390/foods14203537/s1, Supplementary Material S1: Raw dataset of bodyweight, blood pressure, organ index and q-PCR; Supplementary Figure S1: Individual BP data for each rat evaluated; Supplementary Figure S2: Changes in blood pressure (Δ) in SHRs after supplementation; Supplementary Table S1: Effect on blood pressure in SHRs supplemented with buffer BIS-TRIS Propane (n = 7) (1 mL, 20 mM) and optimized chickpea hydrolysate (n = 7) (50 mg/kg of body weight); Supplementary Figure S3: Correlation between relative gene expression levels of ACE2 and Mas1 with MBP and DBP in SHRs supplemented with buffer BIS-TRIS Propane (n = 5) (1 mL, 20 mM) and optimized chickpea hydrolysate (n = 5) (50 mg/kg of body weight).

## Abbreviations

The following abbreviations are used in this manuscript:

ACE1	Angiotensin-I-converting enzyme
BP	Blood pressure
SHRs	Spontaneously hypertensive rats
OCPH	Optimized chickpea protein hydrolysate
RAAS	Renin–angiotensin–aldosterone system
AngII	Angiotensin II
AT_1_R	Angiotensin type 1 receptor
Mas1	G protein-coupled Mas receptor
SBP	Systolic blood pressure
DBP	Diastolic blood pressure
MBP	Mean blood pressure

## References

[1] Mills K.T., Stefanescu A., He J. (2020). The Global Epidemiology of Hypertension. Nat. Rev. Nephrol..

[2] Brouwers S., Sudano I., Kokubo Y., Sulaica E.M. (2021). Arterial Hypertension. Lancet.

[3] Valenzuela P.L., Carrera-Bastos P., Gálvez B.G., Ruiz-Hurtado G., Ordovas J.M., Ruilope L.M., Lucia A. (2021). Lifestyle Interventions for the Prevention and Treatment of Hypertension. Nat. Rev. Cardiol..

[4] Carey R.M., Moran A.E., Whelton P.K. (2022). Treatment of Hypertension: A Review. JAMA.

[5] Albasri A., Hattle M., Koshiaris C., Dunnigan A., Paxton B., Fox S.E., Smith M., Archer L., Levis B., Payne R.A. (2021). Association between Antihypertensive Treatment and Adverse Events: Systematic Review and Meta-Analysis. BMJ.

[6] Na Takuathung M., Sakuludomkan W., Khatsri R., Dukaew N., Kraivisitkul N., Ahmadmusa B., Mahakkanukrauh C., Wangthaweesap K., Onin J., Srichai S. (2022). Adverse Effects of Angiotensin-Converting Enzyme Inhibitors in Humans: A Systematic Review and Meta-Analysis of 378 Randomized Controlled Trials. Int. J. Environ. Res. Public. Health.

[7] Kaur A., Kehinde B.A., Sharma P., Sharma D., Kaur S. (2021). Recently Isolated Food-Derived Antihypertensive Hydrolysates and Peptides: A Review. Food Chem..

[8] Olalere O.A., Yap P.-G., Gan C.-Y. (2023). Comprehensive Review on Some Food-Derived Bioactive Peptides with Anti-Hypertension Therapeutic Potential for Angiotensin-Converting Enzyme (ACE) Inhibition. J. Proteins Proteom..

[9] Carbonaro M., Nucara A. (2022). Legume Proteins and Peptides as Compounds in Nutraceuticals: A Structural Basis for Dietary Health Effects. Nutrients.

[10] Tawalbeh D., Al-U’datt M.H., Wan Ahmad W.A.N., Ahmad F., Sarbon N.M. (2023). Recent Advances in in Vitro and in Vivo Studies of Antioxidant, Ace-Inhibitory and Anti-Inflammatory Peptides from Legume Protein Hydrolysates. Molecules.

[11] Arámburo-Gálvez J.G., Arvizu-Flores A.A., Cárdenas-Torres F.I., Cabrera-Chávez F., Ramírez-Torres G.I., Flores-Mendoza L.K., Gastelum-Acosta P.E., Figueroa-Salcido O.G., Ontiveros N. (2022). Prediction of ACE-I Inhibitory Peptides Derived from Chickpea (*Cicer arietinum* L.): In Silico Assessments Using Simulated Enzymatic Hydrolysis, Molecular Docking and ADMET Evaluation. Foods.

[12] Chávez-Ontiveros J., Reyes-Moreno C., Ramírez-Torres G.I., Figueroa-Salcido O.G., Arámburo-Gálvez J.G., Montoya-Rodríguez A., Ontiveros N., Cuevas-Rodríguez E.O. (2022). Extrusion Improves the Antihypertensive Potential of a Kabuli Chickpea (*Cicer arietinum* L.) Protein Hydrolysate. Foods.

[13] Figueroa-Salcido O.G., Arámburo-Gálvez J.G., Mora-Melgem J.A., Camacho-Cervantes D.L., Gracia-Valenzuela M.H., Cuevas-Rodríguez E.O., Ontiveros N. (2024). Alcalase-Based Chickpea (*Cicer arietinum* L.) Protein Hydrolysates Efficiently Reduce Systolic Blood Pressure in Spontaneously Hypertensive Rats. Foods.

[14] Mora-Melgem J.A., Arámburo-Gálvez J.G., Cárdenas-Torres F.I., Gonzalez-Santamaria J., Ramírez-Torres G.I., Arvizu-Flores A.A., Figueroa-Salcido O.G., Ontiveros N. (2023). Dipeptidyl Peptidase IV Inhibitory Peptides from Chickpea Proteins (*Cicer arietinum* L.): Pharmacokinetics, Molecular Interactions, and Multi-Bioactivities. Pharmaceuticals.

[15] Triebel H., Castrop H. (2024). The Renin Angiotensin Aldosterone System. Pflüg. Arch.-Eur. J. Physiol..

[16] Colafella K.M.M., Bovée D.M., Danser A.J. (2019). The Renin-Angiotensin-Aldosterone System and Its Therapeutic Targets. Exp. Eye Res..

[17] Povlsen A.L., Grimm D., Wehland M., Infanger M., Krüger M. (2020). The Vasoactive Mas Receptor in Essential Hypertension. J. Clin. Med..

[18] Xiang L., Zheng Z., Guo X., Bai R., Zhao R., Chen H., Qiu Z., Qiao X. (2024). Two Novel Angiotensin I-Converting Enzyme Inhibitory Peptides from Garlic Protein: In Silico Screening, Stability, Antihypertensive Effects in Vivo and Underlying Mechanisms. Food Chem..

[19] He R., Yang Y.-J., Wang Z., Xing C., Yuan J., Wang L.-F., Udenigwe C., Ju X.-R. (2019). Rapeseed Protein-Derived Peptides, LY, RALP, and GHS, Modulates Key Enzymes and Intermediate Products of Renin–Angiotensin System Pathway in Spontaneously Hypertensive Rat. NPJ Sci. Food.

[20] Pan H., She X., Wu H., Ma J., Ren D., Lu J. (2015). Long-Term Regulation of the Local Renin–Angiotensin System in the Myocardium of Spontaneously Hypertensive Rats by Feeding Bioactive Peptides Derived from Spirulina Platensis. J. Agric. Food Chem..

[21] Lu J., Sawano Y., Miyakawa T., Xue Y.-L., Cai M.-Y., Egashira Y., Ren D.-F., Tanokura M. (2011). One-Week Antihypertensive Effect of Ile-Gln-Pro in Spontaneously Hypertensive Rats. J. Agric. Food Chem..

[22] Schmittgen T.D., Livak K.J. (2008). Analyzing Real-Time PCR Data by the Comparative CT Method. Nat. Protoc..

[23] Daliri E.B.-M., Ofosu F.K., Chelliah R., Park M.H., Kim J.-H., Oh D.-H. (2019). Development of a Soy Protein Hydrolysate with an Antihypertensive Effect. Int. J. Mol. Sci..

[24] López-Moreno M., Jiménez-Moreno E., Márquez Gallego A., Vera Pasamontes G., Uranga Ocio J.A., Garcés-Rimón M., Miguel-Castro M. (2023). Red Quinoa Hydrolysates with Antioxidant Properties Improve Cardiovascular Health in Spontaneously Hypertensive Rats. Antioxidants.

[25] Sun X., Wang M., Xu C., Wang S., Li L., Zou S., Yu J., Wei Y. (2022). Positive Effect of a Pea–Clam Two-Peptide Composite on Hypertension and Organ Protection in Spontaneously Hypertensive Rats. Nutrients.

[26] Zhu J., Li J., Guo Y., Quaisie J., Hong C., Ma H. (2021). Antihypertensive and Immunomodulatory Effects of Defatted Corn Germ Hydrolysates: An in Vivo Study. Front. Nutr..

[27] Fan H., Liao W., Spaans F., Pasha M., Davidge S.T., Wu J. (2022). Chicken Muscle Hydrolysate Reduces Blood Pressure in Spontaneously Hypertensive Rats, Upregulates ACE2, and Ameliorates Vascular Inflammation, Fibrosis, and Oxidative Stress. J. Food Sci..

[28] Hultström M. (2012). Development of Structural Kidney Damage in Spontaneously Hypertensive Rats. J. Hypertens..

[29] Bianchi G., Fox U., Di Francesco G., Giovanetti A., Pagetti D. (1974). Blood Pressure Changes Produced by Kidney Cross-Transplantation between Spontaneously Hypertensive Rats and Normotensive Rats. Clin. Sci..

[30] Canoy D., Nazarzadeh M., Copland E., Bidel Z., Rao S., Li Y., Rahimi K. (2022). How Much Lowering of Blood Pressure Is Required to Prevent Cardiovascular Disease in Patients with and without Previous Cardiovascular Disease?. Curr. Cardiol. Rep..

[31] Anishchenko A., Aliev O., Sidekhmenova A., Shamanaev A.Y., Plotnikov M. (2015). Dynamics of Blood Pressure Elevation and Endothelial Dysfunction in SHR Rats during the Development of Arterial Hypertension. Bull. Exp. Biol. Med..

[32] Huang J., Liu Q., Xue B., Chen L., Wang Y., Ou S., Peng X. (2016). Angiotensin-i-Converting Enzyme Inhibitory Activities and in Vivo Antihypertensive Effects of Sardine Protein Hydrolysate. J. Food Sci..

[33] Li H., Prairie N., Udenigwe C.C., Adebiyi A.P., Tappia P.S., Aukema H.M., Jones P.J., Aluko R.E. (2011). Blood Pressure Lowering Effect of a Pea Protein Hydrolysate in Hypertensive Rats and Humans. J. Agric. Food Chem..

[34] Mas-Capdevila A., Pons Z., Aleixandre A., Bravo F.I., Muguerza B. (2018). Dose-Related Antihypertensive Properties and the Corresponding Mechanisms of a Chicken Foot Hydrolysate in Hypertensive Rats. Nutrients.

[35] Fritz M., Vecchi B., Rinaldi G., Añón M.C. (2011). Amaranth Seed Protein Hydrolysates Have in Vivo and in Vitro Antihyperten-sive Activity. Food Chem..

[36] Liao W., Fan H., Davidge S.T., Wu J. (2019). Egg White–Derived Antihypertensive Peptide IRW (Ile-Arg-Trp) Reduces Blood Pressure in Spontaneously Hypertensive Rats via the ACE2/Ang (1-7)/Mas Receptor Axis. Mol. Nutr. Food Res..

[37] Jahandideh F., Chakrabarti S., Majumder K., Li Q., Panahi S., Morton J.S., Davidge S.T., Wu J. (2016). Egg White Protein Hydrolysate Reduces Blood Pressure, Improves Vascular Relaxation and Modifies Aortic Angiotensin II Receptors Expression in Spontaneously Hypertensive Rats. J. Funct. Foods.

[38] Yu Z., Yin Y., Zhao W., Chen F., Liu J. (2014). Antihypertensive Effect of Angiotensin-Converting Enzyme Inhibitory Peptide RVPSL on Spontaneously Hypertensive Rats by Regulating Gene Expression of the Renin–Angiotensin System. J. Agric. Food Chem..

[39] Janhavi P., Divyashree S., Sanjailal K., Muthukumar S. (2022). DoseCal: A Virtual Calculator for Dosage Conversion between Human and Different Animal Species. Arch. Physiol. Biochem..

[40] Wang Z., Fan H., Bao X., Wu J. (2023). Angiotensin-Converting Enzyme 2 Activation Is Not a Common Feature of Angiotensin-Converting Enzyme Inhibitory Peptides. J. Agric. Food Chem..

[41] Chappell M.C. (2007). Emerging Evidence for a Functional Angiotensin-Converting Enzyme 2-Angiotensin-(1-7)-MAS Receptor Axis: More than Regulation of Blood Pressure?. Hypertension.

[42] Koka V., Huang X.R., Chung A.C., Wang W., Truong L.D., Lan H.Y. (2008). Angiotensin II Up-Regulates Angiotensin I-Converting Enzyme (ACE), but down-Regulates ACE2 via the AT1-ERK/P38 MAP Kinase Pathway. Am. J. Pathol..

[43] Carey R.M. (2004). Angiotensin Type-1 Receptor Blockade Increases ACE 2 Expression in the Heart. Hypertension.

[44] Coronado-Cáceres L.J., Hernández-Ledesma B., Mojica L., Quevedo-Corona L., Rabadán-Chávez G., Castillo-Herrera G.A., Lugo Cervantes E. (2021). Cocoa (*Theobroma cacao* L.) Seed-Derived Peptides Reduce Blood Pressure by Interacting with the Catalytic Site of the Angiotensin-Converting Enzyme. Foods.

[45] Fadimu G.J., Gan C.-Y., Olalere O.A., Farahnaky A., Gill H., Truong T. (2023). Novel Antihypertensive Peptides from Lupin Protein Hydrolysate: An in-Silico Identification and Molecular Docking Studies. Food Chem..

