# Supplementary material for: An Optimized Chickpea Protein Hydrolysate Exerts Long-Term Antihypertensive Effects and Upregulates ACE2 and Mas1 Gene Expression in Spontaneously Hypertensive Rats
Source: Foods. 2025 Oct 17;14(20):3537. doi: 10.3390/foods14203537 (PMC12564324; doi:10.3390/foods14203537)
Supplement: Supplementary file 1 [file foods-14-03537-s001.zip › foods-3899909-supplementary.pdf]

Supplementary material S1. Raw dataset of bodyweight, blood pressure, organ index and q-PCR.

Raw dataset for the manuscript titled "An Optimized Chickpea Protein Hydrolysate Exerts Long-Term Antihypertensive Effects and Upregulates the ACE2/Ang-(1–7)/Mas1 Pathway in Spontaneously Hypertensive Rats," by Oscar Gerardo Figueroa-Salcido, Jesús Gilberto Arámburo-Gálvez, Lilian Karem Flores-Mendoza, Giovanni I. Ramírez-Torres, Martina Hilda Gracia-Valenzuela, Edith Oliva Cuevas-Rodríguez, and Noé Ontiveros.

1. Bodyweight data.

Animal ID	Group	Evaluation week	Results (g)
ID-10	Control	0	250.03
ID-6	Control	0	240.34
ID-5	Control	0	246.76
ID-9	Control	0	232.87
ID-2	Control	0	230.28
ID-3	Control	0	240.44
ID-7	Control	0	261.90
ID-10	Control	1	251.38
ID-6	Control	1	262.52
ID-5	Control	1	258.15
ID-9	Control	1	262.53
ID-2	Control	1	238.07
ID-3	Control	1	244.41
ID-7	Control	1	271.64
ID-10	Control	2	272.18
ID-6	Control	2	264.28
ID-5	Control	2	251.00
ID-9	Control	2	260.00
ID-2	Control	2	248.00
ID-3	Control	2	250.00
ID-7	Control	2	281.06
ID-10	Control	3	284.43
ID-6	Control	3	276.17
ID-5	Control	3	262.30
ID-9	Control	3	271.70
ID-2	Control	3	259.16
ID-3	Control	3	261.25
ID-7	Control	3	293.71
ID-10	Control	4	293.66
ID-6	Control	4	280.80
ID-5	Control	4	265.00
ID-9	Control	4	275.00

Raw dataset for the manuscript titled "An Optimized Chickpea Protein Hydrolysate Exerts Long-Term Antihypertensive Effects and Upregulates the ACE2/Ang-(1–7)/Mas1 Pathway in Spontaneously Hypertensive Rats," by Oscar Gerardo Figueroa-Salcido, Jesús Gilberto Arámburo-Gálvez, Lilian Karem Flores-Mendoza, Giovanni I. Ramírez-Torres, Martina Hilda Gracia-Valenzuela, Edith Oliva Cuevas-Rodríguez, and Noé Ontiveros.

ID-2	Control	4	270.00
ID-3	Control	4	297.50
ID-7	Control	4	300.93
ID-10	Control	5	307.01
ID-6	Control	5	300.23
ID-5	Control	5	267.25
ID-9	Control	5	282.42
ID-2	Control	5	282.42
ID-3	Control	5	298.50
ID-7	Control	5	312.70
ID-13	Intervention	0	245.86
ID-1	Intervention	0	232.87
ID-11	Intervention	0	253.44
ID-12	Intervention	0	246.56
ID-14	Intervention	0	268.77
ID-4	Intervention	0	252.89
ID-8	Intervention	0	206.96
ID-13	Intervention	1	262.53
ID-1	Intervention	1	247.82
ID-11	Intervention	1	264.40
ID-12	Intervention	1	262.40
ID-14	Intervention	1	288.93
ID-4	Intervention	1	267.93
ID-8	Intervention	1	218.23
ID-13	Intervention	2	268.01
ID-1	Intervention	2	258.07
ID-11	Intervention	2	278.90
ID-12	Intervention	2	273.48
ID-14	Intervention	2	290.00
ID-4	Intervention	2	273.00
ID-8	Intervention	2	224.00
ID-13	Intervention	3	278.73
ID-1	Intervention	3	268.39
ID-11	Intervention	3	290.06
ID-12	Intervention	3	284.42
ID-14	Intervention	3	301.60

Raw dataset for the manuscript titled “An Optimized Chickpea Protein Hydrolysate Exerts Long-Term Antihypertensive Effects and Upregulates the ACE2/Ang-(1–7)/Mas1 Pathway in Spontaneously Hypertensive Rats,” by Oscar Gerardo Figueroa-Salcido, Jesús Gilberto Arámburo-Gálvez, Lilian Karem Flores-Mendoza, Giovanni I. Ramírez-Torres, Martina Hilda Gracia-Valenzuela, Edith Oliva Cuevas-Rodríguez, and Noé Ontiveros.

ID-4	Intervention	3	283.92
ID-8	Intervention	3	232.96
ID-13	Intervention	4	279.80
ID-1	Intervention	4	279.36
ID-11	Intervention	4	293.99
ID-12	Intervention	4	288.71
ID-14	Intervention	4	313.29
ID-4	Intervention	4	297.50
ID-8	Intervention	4	241.28
ID-13	Intervention	5	291.10
ID-1	Intervention	5	293.37
ID-11	Intervention	5	300.37
ID-12	Intervention	5	302.74
ID-14	Intervention	5	320.38
ID-4	Intervention	5	312.22
ID-8	Intervention	5	250.25

Raw dataset for the manuscript titled "An Optimized Chickpea Protein Hydrolysate Exerts Long-Term Antihypertensive Effects and Upregulates the ACE2/Ang-(1–7)/Mas1 Pathway in Spontaneously Hypertensive Rats," by Oscar Gerardo Figueroa-Salcido, Jesús Gilberto Arámburo-Gálvez, Lilian Karem Flores-Mendoza, Giovanni I. Ramírez-Torres, Martina Hilda Gracia-Valenzuela, Edith Oliva Cuevas-Rodríguez, and Noé Ontiveros.

2. Blood Pressure data.

Animal ID	Group	Evaluation day	Blood pressure Component	Results (mmHg)
ID-10	Control	0	Systolic blood pressure	204.10
ID-6	Control	0	Systolic blood pressure	196.25
ID-5	Control	0	Systolic blood pressure	202.09
ID-9	Control	0	Systolic blood pressure	201.33
ID-2	Control	0	Systolic blood pressure	205.00
ID-3	Control	0	Systolic blood pressure	200.00
ID-7	Control	0	Systolic blood pressure	200.89
ID-10	Control	5	Systolic blood pressure	200.83
ID-6	Control	5	Systolic blood pressure	211.83
ID-5	Control	5	Systolic blood pressure	208.00
ID-9	Control	5	Systolic blood pressure	214.17
ID-2	Control	5	Systolic blood pressure	212.85
ID-3	Control	5	Systolic blood pressure	192.11
ID-7	Control	5	Systolic blood pressure	208.66
ID-10	Control	10	Systolic blood pressure	217.57
ID-6	Control	10	Systolic blood pressure	203.18
ID-5	Control	10	Systolic blood pressure	221.80
ID-9	Control	10	Systolic blood pressure	222.25
ID-2	Control	10	Systolic blood pressure	214.50
ID-3	Control	10	Systolic blood pressure	210.16
ID-7	Control	10	Systolic blood pressure	219.22
ID-10	Control	15	Systolic blood pressure	215.42
ID-6	Control	15	Systolic blood pressure	216.60
ID-5	Control	15	Systolic blood pressure	222.25
ID-9	Control	15	Systolic blood pressure	222.42
ID-2	Control	15	Systolic blood pressure	218.20
ID-3	Control	15	Systolic blood pressure	211.75
ID-7	Control	15	Systolic blood pressure	210.10
ID-10	Control	20	Systolic blood pressure	217.28
ID-6	Control	20	Systolic blood pressure	210.00
ID-5	Control	20	Systolic blood pressure	223.50
ID-9	Control	20	Systolic blood pressure	217.54

Raw dataset for the manuscript titled "An Optimized Chickpea Protein Hydrolysate Exerts Long-Term Antihypertensive Effects and Upregulates the ACE2/Ang-(1–7)/Mas1 Pathway in Spontaneously Hypertensive Rats," by Oscar Gerardo Figueroa-Salcido, Jesús Gilberto Arámburo-Gálvez, Lilian Karem Flores-Mendoza, Giovanni I. Ramírez-Torres, Martina Hilda Gracia-Valenzuela, Edith Oliva Cuevas-Rodríguez, and Noé Ontiveros.

ID-2	Control	20	Systolic blood pressure	218.46
ID-3	Control	20	Systolic blood pressure	219.55
ID-7	Control	20	Systolic blood pressure	211.50
ID-10	Control	25	Systolic blood pressure	228.27
ID-6	Control	25	Systolic blood pressure	203.33
ID-5	Control	25	Systolic blood pressure	210.00
ID-9	Control	25	Systolic blood pressure	214.80
ID-2	Control	25	Systolic blood pressure	218.16
ID-3	Control	25	Systolic blood pressure	220.11
ID-7	Control	25	Systolic blood pressure	207.50
ID-10	Control	30	Systolic blood pressure	221.66
ID-6	Control	30	Systolic blood pressure	206.16
ID-5	Control	30	Systolic blood pressure	211.40
ID-9	Control	30	Systolic blood pressure	222.30
ID-2	Control	30	Systolic blood pressure	211.20
ID-3	Control	30	Systolic blood pressure	225.66
ID-7	Control	30	Systolic blood pressure	203.50
ID-10	Control	35	Systolic blood pressure	223.83
ID-6	Control	35	Systolic blood pressure	206.66
ID-5	Control	35	Systolic blood pressure	220.25
ID-9	Control	35	Systolic blood pressure	227.00
ID-2	Control	35	Systolic blood pressure	215.16
ID-3	Control	35	Systolic blood pressure	205.00
ID-7	Control	35	Systolic blood pressure	208.60
ID-13	Intervention	0	Systolic blood pressure	205.40
ID-1	Intervention	0	Systolic blood pressure	206.27
ID-11	Intervention	0	Systolic blood pressure	197.88
ID-12	Intervention	0	Systolic blood pressure	208.57
ID-14	Intervention	0	Systolic blood pressure	196.13
ID-4	Intervention	0	Systolic blood pressure	198.28
ID-8	Intervention	0	Systolic blood pressure	199.50
ID-13	Intervention	5	Systolic blood pressure	202.50
ID-1	Intervention	5	Systolic blood pressure	191.80
ID-11	Intervention	5	Systolic blood pressure	199.77
ID-12	Intervention	5	Systolic blood pressure	183.33
ID-14	Intervention	5	Systolic blood pressure	188.88

Raw dataset for the manuscript titled "An Optimized Chickpea Protein Hydrolysate Exerts Long-Term Antihypertensive Effects and Upregulates the ACE2/Ang-(1–7)/Mas1 Pathway in Spontaneously Hypertensive Rats," by Oscar Gerardo Figueroa-Salcido, Jesús Gilberto Arámburo-Gálvez, Lilian Karem Flores-Mendoza, Giovanni I. Ramírez-Torres, Martina Hilda Gracia-Valenzuela, Edith Oliva Cuevas-Rodríguez, and Noé Ontiveros.

ID-4	Intervention	5	Systolic blood pressure	188.08
ID-8	Intervention	5	Systolic blood pressure	191.12
ID-13	Intervention	10	Systolic blood pressure	195.75
ID-1	Intervention	10	Systolic blood pressure	176.00
ID-11	Intervention	10	Systolic blood pressure	190.77
ID-12	Intervention	10	Systolic blood pressure	205.55
ID-14	Intervention	10	Systolic blood pressure	198.00
ID-4	Intervention	10	Systolic blood pressure	187.70
ID-8	Intervention	10	Systolic blood pressure	182.80
ID-13	Intervention	15	Systolic blood pressure	200.57
ID-1	Intervention	15	Systolic blood pressure	176.52
ID-11	Intervention	15	Systolic blood pressure	184.40
ID-12	Intervention	15	Systolic blood pressure	190.55
ID-14	Intervention	15	Systolic blood pressure	175.85
ID-4	Intervention	15	Systolic blood pressure	182.20
ID-8	Intervention	15	Systolic blood pressure	181.37
ID-13	Intervention	20	Systolic blood pressure	182.80
ID-1	Intervention	20	Systolic blood pressure	182.20
ID-11	Intervention	20	Systolic blood pressure	194.00
ID-12	Intervention	20	Systolic blood pressure	186.45
ID-14	Intervention	20	Systolic blood pressure	186.37
ID-4	Intervention	20	Systolic blood pressure	186.50
ID-8	Intervention	20	Systolic blood pressure	180.71
ID-13	Intervention	25	Systolic blood pressure	180.00
ID-1	Intervention	25	Systolic blood pressure	181.62
ID-11	Intervention	25	Systolic blood pressure	185.25
ID-12	Intervention	25	Systolic blood pressure	176.00
ID-14	Intervention	25	Systolic blood pressure	179.66
ID-4	Intervention	25	Systolic blood pressure	190.00
ID-8	Intervention	25	Systolic blood pressure	185.80
ID-13	Intervention	30	Systolic blood pressure	182.57
ID-1	Intervention	30	Systolic blood pressure	177.25
ID-11	Intervention	30	Systolic blood pressure	183.77
ID-12	Intervention	30	Systolic blood pressure	179.83
ID-14	Intervention	30	Systolic blood pressure	187.60
ID-4	Intervention	30	Systolic blood pressure	187.80

Raw dataset for the manuscript titled "An Optimized Chickpea Protein Hydrolysate Exerts Long-Term Antihypertensive Effects and Upregulates the ACE2/Ang-(1–7)/Mas1 Pathway in Spontaneously Hypertensive Rats," by Oscar Gerardo Figueroa-Salcido, Jesús Gilberto Arámburo-Gálvez, Lilian Karem Flores-Mendoza, Giovanni I. Ramírez-Torres, Martina Hilda Gracia-Valenzuela, Edith Oliva Cuevas-Rodríguez, and Noé Ontiveros.

ID-8	Intervention	30	Systolic blood pressure	188.70
ID-13	Intervention	35	Systolic blood pressure	181.23
ID-1	Intervention	35	Systolic blood pressure	174.77
ID-11	Intervention	35	Systolic blood pressure	184.75
ID-12	Intervention	35	Systolic blood pressure	185.20
ID-14	Intervention	35	Systolic blood pressure	165.66
ID-4	Intervention	35	Systolic blood pressure	188.75
ID-8	Intervention	35	Systolic blood pressure	187.16
ID-10	Control	0	Diastolic blood pressure	148.20
ID-6	Control	0	Diastolic blood pressure	143.33
ID-5	Control	0	Diastolic blood pressure	153.50
ID-9	Control	0	Diastolic blood pressure	149.60
ID-2	Control	0	Diastolic blood pressure	143.57
ID-3	Control	0	Diastolic blood pressure	141.25
ID-7	Control	0	Diastolic blood pressure	146.00
ID-10	Control	5	Diastolic blood pressure	154.33
ID-6	Control	5	Diastolic blood pressure	154.83
ID-5	Control	5	Diastolic blood pressure	155.76
ID-9	Control	5	Diastolic blood pressure	163.46
ID-2	Control	5	Diastolic blood pressure	150.57
ID-3	Control	5	Diastolic blood pressure	136.77
ID-7	Control	5	Diastolic blood pressure	147.83
ID-10	Control	10	Diastolic blood pressure	162.07
ID-6	Control	10	Diastolic blood pressure	150.28
ID-5	Control	10	Diastolic blood pressure	169.45
ID-9	Control	10	Diastolic blood pressure	175.00
ID-2	Control	10	Diastolic blood pressure	157.33
ID-3	Control	10	Diastolic blood pressure	156.33
ID-7	Control	10	Diastolic blood pressure	162.66
ID-10	Control	15	Diastolic blood pressure	172.14
ID-6	Control	15	Diastolic blood pressure	163.40
ID-5	Control	15	Diastolic blood pressure	174.62
ID-9	Control	15	Diastolic blood pressure	162.57
ID-2	Control	15	Diastolic blood pressure	168.12
ID-3	Control	15	Diastolic blood pressure	157.12
ID-7	Control	15	Diastolic blood pressure	155.90

Raw dataset for the manuscript titled "An Optimized Chickpea Protein Hydrolysate Exerts Long-Term Antihypertensive Effects and Upregulates the ACE2/Ang-(1–7)/Mas1 Pathway in Spontaneously Hypertensive Rats," by Oscar Gerardo Figueroa-Salcido, Jesús Gilberto Arámburo-Gálvez, Lilian Karem Flores-Mendoza, Giovanni I. Ramírez-Torres, Martina Hilda Gracia-Valenzuela, Edith Oliva Cuevas-Rodríguez, and Noé Ontiveros.

ID-10	Control	20	Diastolic blood pressure	169.33
ID-6	Control	20	Diastolic blood pressure	151.83
ID-5	Control	20	Diastolic blood pressure	177.75
ID-9	Control	20	Diastolic blood pressure	166.18
ID-2	Control	20	Diastolic blood pressure	166.00
ID-3	Control	20	Diastolic blood pressure	166.11
ID-7	Control	20	Diastolic blood pressure	167.00
ID-10	Control	25	Diastolic blood pressure	179.54
ID-6	Control	25	Diastolic blood pressure	140.66
ID-5	Control	25	Diastolic blood pressure	155.00
ID-9	Control	25	Diastolic blood pressure	163.40
ID-2	Control	25	Diastolic blood pressure	158.85
ID-3	Control	25	Diastolic blood pressure	161.11
ID-7	Control	25	Diastolic blood pressure	153.50
ID-10	Control	30	Diastolic blood pressure	172.33
ID-6	Control	30	Diastolic blood pressure	155.66
ID-5	Control	30	Diastolic blood pressure	144.60
ID-9	Control	30	Diastolic blood pressure	178.00
ID-2	Control	30	Diastolic blood pressure	155.60
ID-3	Control	30	Diastolic blood pressure	177.00
ID-7	Control	30	Diastolic blood pressure	151.83
ID-10	Control	35	Diastolic blood pressure	161.33
ID-6	Control	35	Diastolic blood pressure	155.16
ID-5	Control	35	Diastolic blood pressure	167.75
ID-9	Control	35	Diastolic blood pressure	170.66
ID-2	Control	35	Diastolic blood pressure	165.00
ID-3	Control	35	Diastolic blood pressure	149.60
ID-7	Control	35	Diastolic blood pressure	145.60
ID-13	Intervention	0	Diastolic blood pressure	159.20
ID-1	Intervention	0	Diastolic blood pressure	143.63
ID-11	Intervention	0	Diastolic blood pressure	142.62
ID-12	Intervention	0	Diastolic blood pressure	158.14
ID-14	Intervention	0	Diastolic blood pressure	133.00
ID-4	Intervention	0	Diastolic blood pressure	140.57
ID-8	Intervention	0	Diastolic blood pressure	130.62
ID-13	Intervention	5	Diastolic blood pressure	145.60

Raw dataset for the manuscript titled "An Optimized Chickpea Protein Hydrolysate Exerts Long-Term Antihypertensive Effects and Upregulates the ACE2/Ang-(1–7)/Mas1 Pathway in Spontaneously Hypertensive Rats," by Oscar Gerardo Figueroa-Salcido, Jesús Gilberto Arámburo-Gálvez, Lilian Karem Flores-Mendoza, Giovanni I. Ramírez-Torres, Martina Hilda Gracia-Valenzuela, Edith Oliva Cuevas-Rodríguez, and Noé Ontiveros.

ID-1	Intervention	5	Diastolic blood pressure	139.90
ID-11	Intervention	5	Diastolic blood pressure	146.11
ID-12	Intervention	5	Diastolic blood pressure	125.50
ID-14	Intervention	5	Diastolic blood pressure	130.00
ID-4	Intervention	5	Diastolic blood pressure	136.81
ID-8	Intervention	5	Diastolic blood pressure	126.33
ID-13	Intervention	10	Diastolic blood pressure	135.66
ID-1	Intervention	10	Diastolic blood pressure	119.90
ID-11	Intervention	10	Diastolic blood pressure	133.00
ID-12	Intervention	10	Diastolic blood pressure	153.28
ID-14	Intervention	10	Diastolic blood pressure	143.85
ID-4	Intervention	10	Diastolic blood pressure	133.70
ID-8	Intervention	10	Diastolic blood pressure	121.20
ID-13	Intervention	15	Diastolic blood pressure	141.85
ID-1	Intervention	15	Diastolic blood pressure	116.90
ID-11	Intervention	15	Diastolic blood pressure	127.80
ID-12	Intervention	15	Diastolic blood pressure	114.00
ID-14	Intervention	15	Diastolic blood pressure	111.28
ID-4	Intervention	15	Diastolic blood pressure	120.30
ID-8	Intervention	15	Diastolic blood pressure	113.75
ID-13	Intervention	20	Diastolic blood pressure	130.80
ID-1	Intervention	20	Diastolic blood pressure	132.10
ID-11	Intervention	20	Diastolic blood pressure	143.85
ID-12	Intervention	20	Diastolic blood pressure	133.27
ID-14	Intervention	20	Diastolic blood pressure	128.37
ID-4	Intervention	20	Diastolic blood pressure	141.50
ID-8	Intervention	20	Diastolic blood pressure	120.14
ID-13	Intervention	25	Diastolic blood pressure	124.33
ID-1	Intervention	25	Diastolic blood pressure	120.12
ID-11	Intervention	25	Diastolic blood pressure	123.66
ID-12	Intervention	25	Diastolic blood pressure	121.00
ID-14	Intervention	25	Diastolic blood pressure	120.83
ID-4	Intervention	25	Diastolic blood pressure	118.80
ID-8	Intervention	25	Diastolic blood pressure	130.00
ID-13	Intervention	30	Diastolic blood pressure	123.28
ID-1	Intervention	30	Diastolic blood pressure	119.36

Raw dataset for the manuscript titled "An Optimized Chickpea Protein Hydrolysate Exerts Long-Term Antihypertensive Effects and Upregulates the ACE2/Ang-(1–7)/Mas1 Pathway in Spontaneously Hypertensive Rats," by Oscar Gerardo Figueroa-Salcido, Jesús Gilberto Arámburo-Gálvez, Lilian Karem Flores-Mendoza, Giovanni I. Ramírez-Torres, Martina Hilda Gracia-Valenzuela, Edith Oliva Cuevas-Rodríguez, and Noé Ontiveros.

ID-11	Intervention	30	Diastolic blood pressure	140.33
ID-12	Intervention	30	Diastolic blood pressure	128.50
ID-14	Intervention	30	Diastolic blood pressure	142.00
ID-4	Intervention	30	Diastolic blood pressure	137.20
ID-8	Intervention	30	Diastolic blood pressure	135.00
ID-13	Intervention	35	Diastolic blood pressure	138.00
ID-1	Intervention	35	Diastolic blood pressure	116.85
ID-11	Intervention	35	Diastolic blood pressure	128.16
ID-12	Intervention	35	Diastolic blood pressure	135.00
ID-14	Intervention	35	Diastolic blood pressure	105.00
ID-4	Intervention	35	Diastolic blood pressure	118.00
ID-8	Intervention	35	Diastolic blood pressure	122.16
ID-10	Control	0	Mean blood pressure	166.40
ID-6	Control	0	Mean blood pressure	160.75
ID-5	Control	0	Mean blood pressure	167.10
ID-9	Control	0	Mean blood pressure	164.77
ID-2	Control	0	Mean blood pressure	163.85
ID-3	Control	0	Mean blood pressure	160.75
ID-7	Control	0	Mean blood pressure	164.55
ID-10	Control	5	Mean blood pressure	169.50
ID-6	Control	5	Mean blood pressure	173.50
ID-5	Control	5	Mean blood pressure	172.84
ID-9	Control	5	Mean blood pressure	180.23
ID-2	Control	5	Mean blood pressure	171.14
ID-3	Control	5	Mean blood pressure	154.77
ID-7	Control	5	Mean blood pressure	167.75
ID-10	Control	10	Mean blood pressure	180.21
ID-6	Control	10	Mean blood pressure	167.71
ID-5	Control	10	Mean blood pressure	186.18
ID-9	Control	10	Mean blood pressure	190.50
ID-2	Control	10	Mean blood pressure	176.00
ID-3	Control	10	Mean blood pressure	174.16
ID-7	Control	10	Mean blood pressure	181.11
ID-10	Control	15	Mean blood pressure	186.42
ID-6	Control	15	Mean blood pressure	180.60
ID-5	Control	15	Mean blood pressure	190.50

Raw dataset for the manuscript titled "An Optimized Chickpea Protein Hydrolysate Exerts Long-Term Antihypertensive Effects and Upregulates the ACE2/Ang-(1–7)/Mas1 Pathway in Spontaneously Hypertensive Rats," by Oscar Gerardo Figueroa-Salcido, Jesús Gilberto Arámburo-Gálvez, Lilian Karem Flores-Mendoza, Giovanni I. Ramírez-Torres, Martina Hilda Gracia-Valenzuela, Edith Oliva Cuevas-Rodríguez, and Noé Ontiveros.

ID-9	Control	15	Mean blood pressure	182.14
ID-2	Control	15	Mean blood pressure	184.50
ID-3	Control	15	Mean blood pressure	175.00
ID-7	Control	15	Mean blood pressure	172.20
ID-10	Control	20	Mean blood pressure	185.25
ID-6	Control	20	Mean blood pressure	170.50
ID-5	Control	20	Mean blood pressure	192.75
ID-9	Control	20	Mean blood pressure	183.00
ID-2	Control	20	Mean blood pressure	183.15
ID-3	Control	20	Mean blood pressure	183.66
ID-7	Control	20	Mean blood pressure	181.66
ID-10	Control	25	Mean blood pressure	195.45
ID-6	Control	25	Mean blood pressure	161.33
ID-5	Control	25	Mean blood pressure	173.00
ID-9	Control	25	Mean blood pressure	180.40
ID-2	Control	25	Mean blood pressure	178.28
ID-3	Control	25	Mean blood pressure	180.55
ID-7	Control	25	Mean blood pressure	171.25
ID-10	Control	30	Mean blood pressure	188.66
ID-6	Control	30	Mean blood pressure	172.00
ID-5	Control	30	Mean blood pressure	166.40
ID-9	Control	30	Mean blood pressure	192.50
ID-2	Control	30	Mean blood pressure	173.60
ID-3	Control	30	Mean blood pressure	193.50
ID-7	Control	30	Mean blood pressure	168.50
ID-10	Control	35	Mean blood pressure	181.33
ID-6	Control	35	Mean blood pressure	172.00
ID-5	Control	35	Mean blood pressure	185.00
ID-9	Control	35	Mean blood pressure	183.00
ID-2	Control	35	Mean blood pressure	181.50
ID-3	Control	35	Mean blood pressure	167.00
ID-7	Control	35	Mean blood pressure	166.20
ID-13	Intervention	0	Mean blood pressure	174.40
ID-1	Intervention	0	Mean blood pressure	164.27
ID-11	Intervention	0	Mean blood pressure	160.75
ID-12	Intervention	0	Mean blood pressure	174.57

Raw dataset for the manuscript titled "An Optimized Chickpea Protein Hydrolysate Exerts Long-Term Antihypertensive Effects and Upregulates the ACE2/Ang-(1–7)/Mas1 Pathway in Spontaneously Hypertensive Rats," by Oscar Gerardo Figueroa-Salcido, Jesús Gilberto Arámburo-Gálvez, Lilian Karem Flores-Mendoza, Giovanni I. Ramírez-Torres, Martina Hilda Gracia-Valenzuela, Edith Oliva Cuevas-Rodríguez, and Noé Ontiveros.

ID-14	Intervention	0	Mean blood pressure	153.62
ID-4	Intervention	0	Mean blood pressure	159.42
ID-8	Intervention	0	Mean blood pressure	154.12
ID-13	Intervention	5	Mean blood pressure	164.20
ID-1	Intervention	5	Mean blood pressure	156.90
ID-11	Intervention	5	Mean blood pressure	163.66
ID-12	Intervention	5	Mean blood pressure	144.50
ID-14	Intervention	5	Mean blood pressure	149.16
ID-4	Intervention	5	Mean blood pressure	153.36
ID-8	Intervention	5	Mean blood pressure	147.66
ID-13	Intervention	10	Mean blood pressure	153.66
ID-1	Intervention	10	Mean blood pressure	138.30
ID-11	Intervention	10	Mean blood pressure	153.42
ID-12	Intervention	10	Mean blood pressure	171.85
ID-14	Intervention	10	Mean blood pressure	161.57
ID-4	Intervention	10	Mean blood pressure	151.40
ID-8	Intervention	10	Mean blood pressure	141.40
ID-13	Intervention	15	Mean blood pressure	161.28
ID-1	Intervention	15	Mean blood pressure	136.50
ID-11	Intervention	15	Mean blood pressure	146.40
ID-12	Intervention	15	Mean blood pressure	139.33
ID-14	Intervention	15	Mean blood pressure	132.32
ID-4	Intervention	15	Mean blood pressure	140.60
ID-8	Intervention	15	Mean blood pressure	136.00
ID-13	Intervention	20	Mean blood pressure	147.80
ID-1	Intervention	20	Mean blood pressure	148.40
ID-11	Intervention	20	Mean blood pressure	159.85
ID-12	Intervention	20	Mean blood pressure	150.63
ID-14	Intervention	20	Mean blood pressure	147.37
ID-4	Intervention	20	Mean blood pressure	156.00
ID-8	Intervention	20	Mean blood pressure	140.28
ID-13	Intervention	25	Mean blood pressure	143.33
ID-1	Intervention	25	Mean blood pressure	140.25
ID-11	Intervention	25	Mean blood pressure	152.00
ID-12	Intervention	25	Mean blood pressure	138.80
ID-14	Intervention	25	Mean blood pressure	140.33

Raw dataset for the manuscript titled "An Optimized Chickpea Protein Hydrolysate Exerts Long-Term Antihypertensive Effects and Upregulates the ACE2/Ang-(1–7)/Mas1 Pathway in Spontaneously Hypertensive Rats," by Oscar Gerardo Figueroa-Salcido, Jesús Gilberto Arámburo-Gálvez, Lilian Karem Flores-Mendoza, Giovanni I. Ramírez-Torres, Martina Hilda Gracia-Valenzuela, Edith Oliva Cuevas-Rodríguez, and Noé Ontiveros.

ID-4	Intervention	25	Mean blood pressure	138.20
ID-8	Intervention	25	Mean blood pressure	148.40
ID-13	Intervention	30	Mean blood pressure	142.71
ID-1	Intervention	30	Mean blood pressure	138.27
ID-11	Intervention	30	Mean blood pressure	154.40
ID-12	Intervention	30	Mean blood pressure	145.33
ID-14	Intervention	30	Mean blood pressure	156.80
ID-4	Intervention	30	Mean blood pressure	153.60
ID-8	Intervention	30	Mean blood pressure	152.50
ID-13	Intervention	35	Mean blood pressure	152.00
ID-1	Intervention	35	Mean blood pressure	134.50
ID-11	Intervention	35	Mean blood pressure	148.33
ID-12	Intervention	35	Mean blood pressure	151.60
ID-14	Intervention	35	Mean blood pressure	124.80
ID-4	Intervention	35	Mean blood pressure	141.50
ID-8	Intervention	35	Mean blood pressure	143.50

Raw dataset for the manuscript titled “An Optimized Chickpea Protein Hydrolysate Exerts Long-Term Antihypertensive Effects and Upregulates the ACE2/Ang-(1–7)/Mas1 Pathway in Spontaneously Hypertensive Rats,” by Oscar Gerardo Figueroa-Salcido, Jesús Gilberto Arámburo-Gálvez, Lilian Karem Flores-Mendoza, Giovanni I. Ramírez-Torres, Martina Hilda Gracia-Valenzuela, Edith Oliva Cuevas-Rodríguez, and Noé Ontiveros.

3. Organ Index data.

Animal ID	Group	Organ	Organ Index
ID-10	Control	Heart	0.436
ID-6	Control	Heart	0.443
ID-5	Control	Heart	0.406
ID-9	Control	Heart	0.479
ID-2	Control	Heart	0.510
ID-2	Control	Heart	0.460
ID-7	Control	Heart	0.482
ID-13	Intervention	Heart	0.386
ID-1	Intervention	Heart	0.459
ID-11	Intervention	Heart	0.468
ID-14	Intervention	Heart	0.449
ID-14	Intervention	Heart	0.463
ID-4	Intervention	Heart	0.395
ID-8	Intervention	Heart	0.400
ID-10	Control	Lung	0.308
ID-6	Control	Lung	0.211
ID-5	Control	Lung	0.235
ID-9	Control	Lung	0.314
ID-2	Control	Lung	0.284
ID-2	Control	Lung	0.249
ID-7	Control	Lung	0.288
ID-13	Intervention	Lung	0.290
ID-1	Intervention	Lung	0.291
ID-11	Intervention	Lung	0.297
ID-14	Intervention	Lung	0.301
ID-14	Intervention	Lung	0.267
ID-4	Intervention	Lung	0.248
ID-8	Intervention	Lung	0.364
ID-10	Control	Spleen	0.164
ID-6	Control	Spleen	0.191
ID-5	Control	Spleen	0.156

Raw dataset for the manuscript titled "An Optimized Chickpea Protein Hydrolysate Exerts Long-Term Antihypertensive Effects and Upregulates the ACE2/Ang-(1–7)/Mas1 Pathway in Spontaneously Hypertensive Rats," by Oscar Gerardo Figueroa-Salcido, Jesús Gilberto Arámburo-Gálvez, Lilian Karem Flores-Mendoza, Giovanni I. Ramírez-Torres, Martina Hilda Gracia-Valenzuela, Edith Oliva Cuevas-Rodríguez, and Noé Ontiveros.

ID-9	Control	Spleen	0.181
ID-2	Control	Spleen	0.185
ID-2	Control	Spleen	0.188
ID-7	Control	Spleen	0.194
ID-13	Intervention	Spleen	0.182
ID-1	Intervention	Spleen	0.169
ID-11	Intervention	Spleen	0.167
ID-14	Intervention	Spleen	0.188
ID-14	Intervention	Spleen	0.186
ID-4	Intervention	Spleen	0.157
ID-8	Intervention	Spleen	0.166
ID-10	Control	Liver	3.593
ID-6	Control	Liver	3.588
ID-5	Control	Liver	3.528
ID-9	Control	Liver	3.864
ID-2	Control	Liver	4.033
ID-2	Control	Liver	3.794
ID-7	Control	Liver	3.869
ID-13	Intervention	Liver	3.195
ID-1	Intervention	Liver	3.943
ID-11	Intervention	Liver	3.985
ID-14	Intervention	Liver	3.883
ID-14	Intervention	Liver	3.319
ID-4	Intervention	Liver	3.667
ID-8	Intervention	Liver	3.307
ID-10	Control	Kidney	0.378
ID-6	Control	Kidney	0.423
ID-5	Control	Kidney	0.384
ID-9	Control	Kidney	0.357
ID-2	Control	Kidney	0.415
ID-2	Control	Kidney	0.411
ID-7	Control	Kidney	0.436
ID-13	Intervention	Kidney	0.350
ID-1	Intervention	Kidney	0.376
ID-11	Intervention	Kidney	0.365
ID-14	Intervention	Kidney	0.371

Raw dataset for the manuscript titled “An Optimized Chickpea Protein Hydrolysate Exerts Long-Term Antihypertensive Effects and Upregulates the ACE2/Ang-(1–7)/Mas1 Pathway in Spontaneously Hypertensive Rats,” by Oscar Gerardo Figueroa-Salcido, Jesús Gilberto Arámburo-Gálvez, Lilian Karem Flores-Mendoza, Giovanni I. Ramírez-Torres, Martina Hilda Gracia-Valenzuela, Edith Oliva Cuevas-Rodríguez, and Noé Ontiveros.

ID-14	Intervention	Kidney	0.358
ID-4	Intervention	Kidney	0.376
ID-8	Intervention	Kidney	0.355

Raw dataset for the manuscript titled "An Optimized Chickpea Protein Hydrolysate Exerts Long-Term Antihypertensive Effects and Upregulates the ACE2/Ang-(1–7)/Mas1 Pathway in Spontaneously Hypertensive Rats," by Oscar Gerardo Figueroa-Salcido, Jesús Gilberto Arámburo-Gálvez, Lilian Karem Flores-Mendoza, Giovanni I. Ramírez-Torres, Martina Hilda Gracia-Valenzuela, Edith Oliva Cuevas-Rodríguez, and Noé Ontiveros.

4. q-PCR data.

Animal ID	Group	Gene	Ct	ΔCt
ID-10	Control	β-actin	23.649	-
ID-6	Control	β-actin	20.699	-
ID-5	Control	β-actin	22.084	-
ID-9	Control	β-actin	22.767	-
ID-3	Control	β-actin	22.691	-
ID-10	Control	Renin	30.375	6.726
ID-6	Control	Renin	28.760	8.061
ID-5	Control	Renin	29.218	7.134
ID-9	Control	Renin	30.929	8.162
ID-3	Control	Renin	30.214	7.524
ID-10	Control	ACE1	34.219	10.570
ID-6	Control	ACE1	28.800	8.101
ID-5	Control	ACE1	30.419	8.335
ID-9	Control	ACE1	32.157	9.390
ID-3	Control	ACE1	32.545	9.855
ID-10	Control	ACE2	32.245	8.596
ID-6	Control	ACE2	29.066	8.367
ID-5	Control	ACE2	32.103	10.019
ID-9	Control	ACE2	31.349	8.582
ID-3	Control	ACE2	31.007	8.317
ID-10	Control	AT1R	30.735	7.086
ID-6	Control	AT1R	28.504	7.805
ID-5	Control	AT1R	29.345	7.261
ID-9	Control	AT1R	30.650	7.883
ID-3	Control	AT1R	30.218	7.524
ID-10	Control	Mas1	33.570	9.921
ID-6	Control	Mas1	31.580	10.881
ID-5	Control	Mas1	31.715	9.631
ID-9	Control	Mas1	33.077	10.310
ID-3	Control	Mas1	32.381	9.690
ID-12	Intervention	β-actin	22.181	-
ID-13	Intervention	β-actin	22.898	-

Raw dataset for the manuscript titled “An Optimized Chickpea Protein Hydrolysate Exerts Long-Term Antihypertensive Effects and Upregulates the ACE2/Ang-(1–7)/Mas1 Pathway in Spontaneously Hypertensive Rats,” by Oscar Gerardo Figueroa-Salcido, Jesús Gilberto Arámburo-Gálvez, Lilian Karem Flores-Mendoza, Giovanni I. Ramírez-Torres, Martina Hilda Gracia-Valenzuela, Edith Oliva Cuevas-Rodríguez, and Noé Ontiveros.

ID-14	Intervention	β-actin	21.861	-
ID-8	Intervention	β-actin	20.980	-
ID-4	Intervention	β-actin	23.107	-
ID-12	Intervention	Renin	29.085	6.905
ID-13	Intervention	Renin	30.720	7.823
ID-14	Intervention	Renin	29.540	7.679
ID-8	Intervention	Renin	28.670	7.691
ID-4	Intervention	Renin	29.667	6.561
ID-12	Intervention	ACE1	31.112	8.932
ID-13	Intervention	ACE1	31.462	8.565
ID-14	Intervention	ACE1	30.295	8.434
ID-8	Intervention	ACE1	30.610	9.630
ID-4	Intervention	ACE1	33.329	10.223
ID-12	Intervention	ACE2	30.585	8.405
ID-13	Intervention	ACE2	30.485	7.588
ID-14	Intervention	ACE2	29.290	7.429
ID-8	Intervention	ACE2	29.056	8.076
ID-4	Intervention	ACE2	31.513	8.407
ID-12	Intervention	AT1R	29.505	7.325
ID-13	Intervention	AT1R	30.395	7.498
ID-14	Intervention	AT1R	29.330	7.469
ID-8	Intervention	AT1R	28.089	7.109
ID-4	Intervention	AT1R	30.176	7.070
ID-12	Intervention	Mas1	31.610	9.430
ID-13	Intervention	Mas1	31.795	8.898
ID-14	Intervention	Mas1	31.940	10.079
ID-8	Intervention	Mas1	30.595	9.616
ID-4	Intervention	Mas1	32.056	8.950

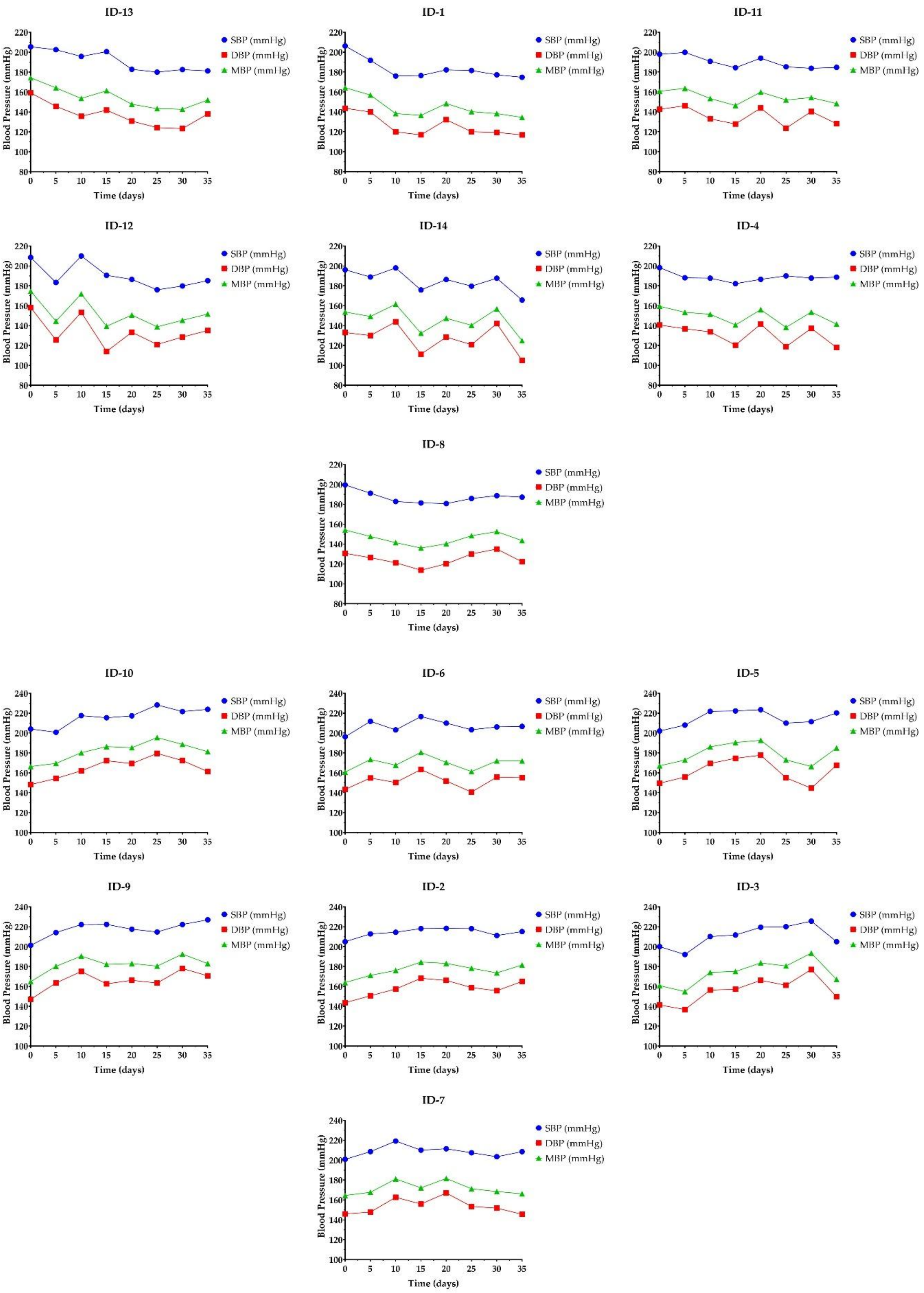

Supplementary Figure S1. Individual BP data for each rat evaluated.

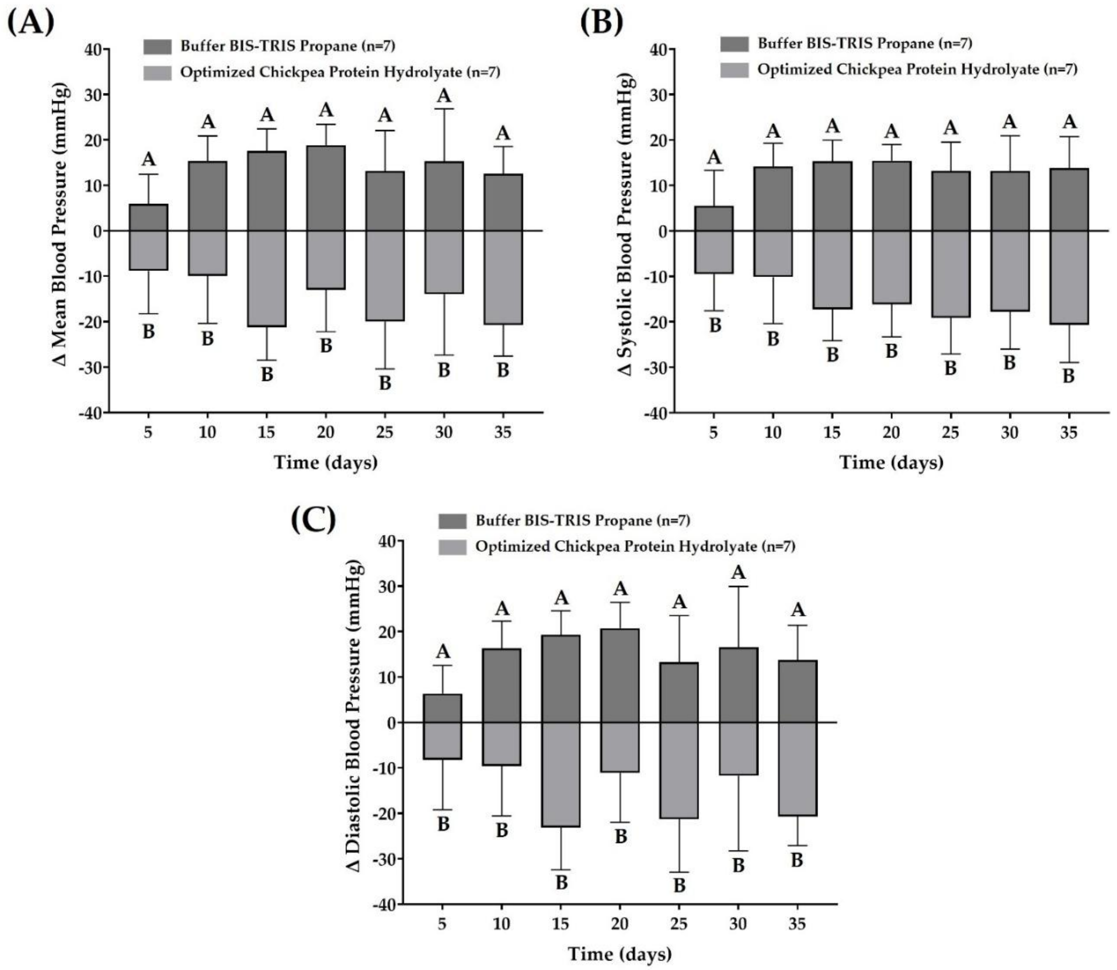

Supplementary Figure S2. Changes in blood pressure (Δ) in SHRs after supplementation with buffer BIS-TRIS Propane (n=7) (1 mL, 20 mM) and optimized chickpea hydrolysate (n=7) (50 mg/kg of body weight). (A) Systolic blood pressure, (B) Diastolic blood pressure, and (C) Mean blood pressure. Data are presented as mean and 95% confidence intervals. Vertically, different letters across treatments indicate statistical differences ($p < 0.05$).

Supplementary Table S1. Effect on blood pressure in SHRs supplemented with buffer BIS-TRIS Propane (n=7) (1 mL, 20 mM) and optimized chickpea hydrolysate (n=7) (50 mg/kg of body weight).

**Intervation**	**Systolic blood pressure**			**Dystolic blood pressure**			**Mean blood pressure**		
**Time (days)**	**BTP (n=7)**	**OCPH (n=7)**	***p value***	**BTP (n=7)**	**OCPH (n=7)**	***p value***	**BTP (n=7)**	**OCPH (n=7)**	***p value***
0	201.4 ± 2.87	201.7 ± 4.90	0.8775	145.6 ± 2.97	144 ± 11.14	0.7207	164 ± 2.49	163 ± 8.66	0.7735
5	206.9 ± 7.87	192.2 ± 6.72	0.0027	151.9 ± 8.26	135.8 ± 8.65	0.0038	170 ± 7.78	154.2 ± 7.74	0.0025
10	215.5 ± 6.88	191.6 ± 11.06	0.0004	161.9 ± 8.32	134.4 ± 11.79	0.0003	179.4 ± 7.62	153.1 ± 11.42	0.0003
15	216.7 ± 4.75	184.5 ± 8.64	<0.0001	164.8 ± 7.14	120.8 ± 10.76	<0.0001	181.6 ± 6.37	141.8 ± 9.65	<0.0001
20	216.8 ± 4.66	185.6 ± 4.39	<0.0001	166.3 ± 7.64	132.9 ± 7.98	<0.0001	182.9 ± 6.56	150 ± 6.35	<0.0001
25	214.6 ± 8.45	182.6 ±4.68	<0.0001	158.9 ± 11.75	122.75 ± 3.76	<0.0001	177.2 ± 10.49	143 ± 5.25	<0.0001
30	214.7 ± 8.63	183.9 ± 4.46	<0.0001	162.1 ± 13.38	132.2 ± 8.69	0.0003	179.3 ± 11.78	149.1 ± 6.97	<0.0001
35	215.2 ± 8.75	181.1 ± 8.20	<0.0001	159.3 ± 9.45	123.3 ± 11.42	<0.0001	176.6 ± 7.95	142.3 ± 9.88	<0.0001

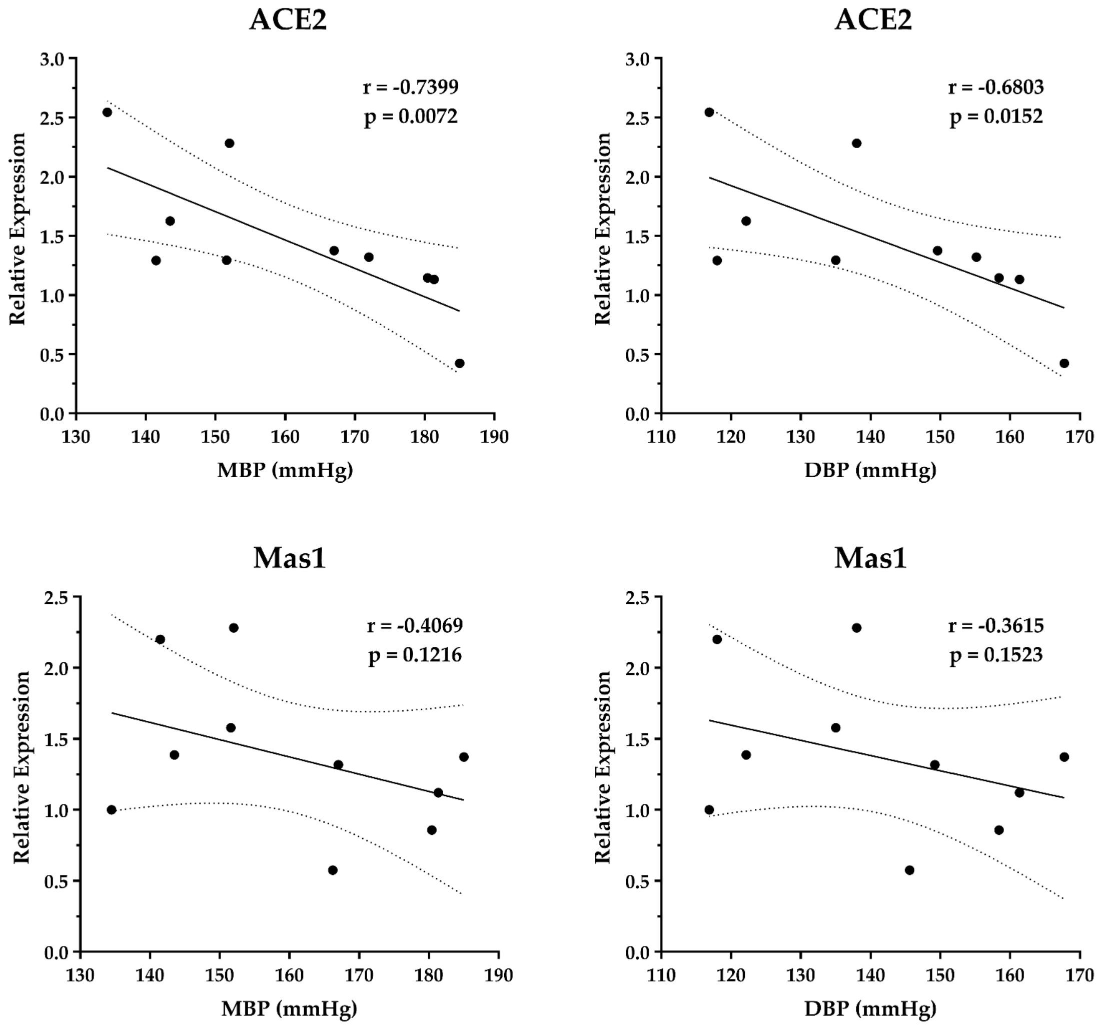

Supplementary Figure S3. Correlation between relative gene expression levels of ACE2 and Mas1 with MBP and DBP in SHRs supplemented with buffer BIS-TRIS Propane (n=5) (1 mL, 20 mM) and optimized chickpea hydrolysate (n=5) (50 mg/kg of body weight). Correlation between relative gene expression and BP was determined using the Pearson's correlation coefficient. A p-value < 0.05 was considered statistically significant.
